# Usefulness of hand grip strength to estimate other physical fitness parameters in older adults

**DOI:** 10.1038/s41598-022-22477-6

**Published:** 2022-10-19

**Authors:** Su Hyun Kim, Taegyu Kim, Jong-Chul Park, Young Hoon Kim

**Affiliations:** 1 Pukyong National University Industry-Cooperation Foundation, 365 Sinseon-ro, Nam-gu, Busan, 48547 Republic of Korea; 2 Department of Marine Sports, Pukyong National University, 45 Youngso-ro, Nam-gu, Busan, 48513 Republic of Korea

**Keywords:** Diagnosis, Geriatrics, Population screening

## Abstract

This study aimed to reveal the status of physical fitness (PF) levels and determine whether hand grip strength (HGS) could be used to estimate other PF parameters in older adults from large population data. A total of 46,269 participants aged ≥ 65 years who participated in the 2019 National Fitness Award Project in South Korea were included in the analysis. Of the participants, 6.8% had the highest level of overall physical fitness, while 48.9% had the lowest level. The proportion of overall PF levels differed significantly according to age groups. Significant associations between HGS and other PF parameters (30-s chair stand test, 2-min or 6-min walk test, sit-and-reach test, 3-m backwards walk test, and Figure-of-8 walk test) were noted and the group with low HGS (< 28 kg for men and < 18 kg for women) had significantly higher odds of having the lowest level of overall PF (odds ratio: 5.232 in men and 6.351 in women), after adjusting for age and body mass index. HGS could estimate muscular strength and endurance, aerobic fitness, flexibility, balance skills, and coordination skills, as well as overall PF level in older adults, and could be used as a substitute test for their PF level in limited situations.

## Introduction

Functional decline usually precedes disability^1^ in older people, and the components of physical fitness (PF) are strongly related to mobility, functional independence, and activities of daily living (ADL)^2^. Therefore, physical functional performance is regarded as the core element for successful aging and quality of life in older people^2^ and assessment of their PF is essential for predicting and preventing frailty and functional disability^3^.

As part of the National Fitness Award (NFA) 100 Project, the Korean Sports Promotion Foundation has developed methods to measure the PF parameters of adolescents^4^, adults^5^ and older adults^6^. They have also reported the age-and sex-matched norms of each parameter to grade the fitness levels of the participants^7^. After measuring each PF parameter, the participants are given a grade of overall PF level according to the grade of each fitness component. Despite 76 measurement centers nationwide, there are still some barriers for testing the PF of older adults, such as accessibility and mobility, impaired cognition, and low fitness level. Unfit individuals may feel tired, have pain during tests^8^, and even have difficulty completing tests. Additionally, PF testing often requires time and space. During the initial stages of the coronavirus disease 2019 (COVID-19) pandemic, testing centers were closed in accordance with the social distancing policy, fearing that close contact among people and production of respiratory droplets during tests would increase the chance of viral transmission.

Among variable PF parameters, hand grip strength (HGS) has been widely used to assess muscular strength of the upper extremity^9,10^ due to its high reliability and simplicity^11–13^, and is also included in the NFA 100 project^6^. Positive correlations between HGS and other lower-limb strength parameters and aerobic capacity have been shown in some studies, although they were based on small sample sizes^14–16^. Due to its relationship with metabolic diseases^17–19^, cardiopulmonary function^20^, cognitive impairment^21^, mental health^22^ and sarcopenia^23^, as well as premature mortality and development of disability^18^, the role of HGS in older adults has been widely emphasized in clinical fields. HGS can be measured readily, even in a narrow space, without causing fatigue and pain or without producing respiratory droplets, as in the measurement of aerobic fitness. Therefore, when PF tests are limited for some reason, if a simple and reliable test such as HGS can estimate the overall PF in healthy older adults, it can be used as a substitute for multiple tests.

This study aimed to reveal the status of Korean older adults’ PF and to determine whether HGS could estimate other PF parameters and overall PF levels of a large population from NFA 100 data.

## Results

Table 1 shows the average values of the baseline characteristics, PF parameters, and proportion of overall PF levels. The participants’ mean age was 72.9 ± 5.6 years. Regarding the overall fitness level, 48.9% of the participants were graded as the 4th, 27.1% as the 3rd, 17.2% as the 2nd, and 6.8% as the 1st level, respectively.

**Table 1:** Characteristics of the participants. Values are presented as mean ± standard deviation or numbers (%).

	Total (n = 46,269)	Men (n = 16,684)	Women (n = 29,585)	P-value
Age (years)	72.9 ± 5.6	73.6 ± 5.5	72.5 ± 5.6	< 0.001
Height (cm)	157.1 ± 8.2	165.0 ± 5.8	152.5 ± 5.4	< 0.001
Weight (kg)	60.8 ± 9.4	66.5 ± 8.9	57.5 ± 7.9	< 0.001
BMI (kg/m^2^)	24.6 ± 3.0	24.4 ± 2.8	24.7 ± 3.1	< 0.001
Fat percent	32.2 ± 19.1	26.5 ± 6.5	35.4 ± 22.7	< 0.001
HGS (kg)	25.9 ± 7.8	33.4 ± 6.4	21.6 ± 4.7	< 0.001
30CST (no.)	19.5 ± 6.3	21.0 ± 6.4	18.6 ± 6.1	< 0.001
6MWT (m)	587.2 ± 93.0	612.2 ± 98.4	577.7 ± 89.1	< 0.001
2MWT (no.)	105.3 ± 25.2	109.1 ± 22.7	103.1 ± 26.3	< 0.001
Sit-and-reach test (cm)	9.8 ± 9.7	3.9 ± 9.6	13.1 ± 8.0	< 0.001
3MBWT (s)	6.4 ± 1.9	6.1 ± 1.6	6.6 ± 2.0	< 0.001
F8WT (s)	26.5 ± 7.0	25.3 ± 6.4	27.2 ± 7.2	< 0.001
**Overall physical fitness level**				
1st (≥ 70th percentile)	3133 (6.8%)	1098 (6.6%)	2035 (6.9%)	< 0.001
2nd (≥ 50th percentile, < 70th percentile)	7966 (17.2%)	2839 (17.0%)	5127 (17.3%)	
3rd (≥ 30th percentile, < 50th percentile)	12,552 (27.1%)	4801 (28.8%)	7751 (26.2%)	
4th (< 30th percentile)	22,618 (48.9%)	7946 (47.6%)	14,672 (49.6%)	

Data were obtained from 2563 participants for 6MWT and 43,716 for 2MWT. P-values from Student’s t-test or χ^2^-test.

*BMI* body mass index, *HGS* hand grip strength, *30CST* 30-s chair stand test, *6MWT* 6-min walk test, *2MWT* 2-min walk test, *3MBWT* 3-m backwards walk test, *F8WT* figure-of-8 walk test.

**Figure 1:**
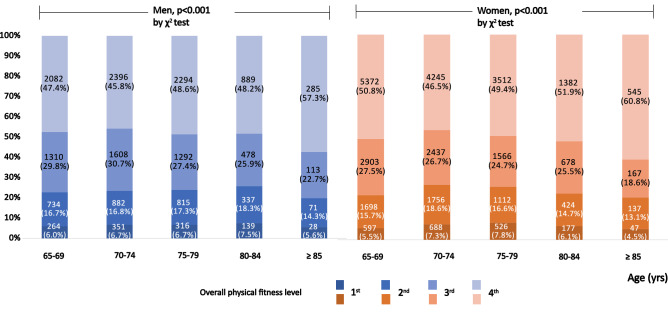
The proportion and significant differences of overall physical fitness level according to the age groups in men and women.

Figure 1 shows the distribution of overall PF levels according to age group. The highest overall fitness was seen in 5.6 to 7.5% of men and 4.5 to 7.8% of women, while the lowest overall fitness was seen in 45.8 to 57.3% of men and 46.5 to 60.8% of women. Significant differences in the proportion of overall PF levels according to age groups (P < 0.001) were seen in both sexes.

HGS showed significantly fair correlations with muscle strength and endurance of the lower limbs [30-s chair stand test (30CST)], aerobic fitness [6-min walk test (6MWT) or 2-min walk test (2MWT)], balance skills [3-m backwards walk test (3MBWT)], and coordination skills [figure-of-8 walk test (F8WT)] (Table 2).

**Table 2:** Correlations between HGS and other physical fitness parameters.

Variables	Pearson’s coefficient,* r* (with HGS, kg)		
	Total	Men	Women
Age (years)	− 0.190***	− 0.379***	− 0.386***
Height (cm)	0.719***	0.408***	0.386***
Weight (kg)	0.519***	0.356***	0.253***
BMI (kg/m^2^)	0.030***	0.164***	0.059***
30CST (no.)	0.350***	0.328***	0.318***
6MWT (m)	0.327***	0.362***	0.306***
2MWT (no.)	0.307***	0.337**	0.337***
Sit-and-reach test (cm)	0.180***	0.210***	0.286***
3MBWT (s)	− 0.350***	− 0.356***	− 0.369***
F8WT (s)	− 0.352***	− 0.375***	− 0.392***

*BMI* body mass index, *HGS* hand grip strength, *30CST* 30-s chair stand test, *6MWT* 6-min walk test, *2MWT* 2-min walk test, *3MBWT* 3-m backwards walk test, *F8WT* figure-of-8 walk test.

***P < 0.001 through Pearson’s correlation analysis.

After adjusting for age and body mass index (BMI), multiple linear regression analysis revealed that HGS was positively associated with the 30CST, 6MWT, 2MWT, and sit-and-reach test, and negatively associated with the 3MBWT and F8WT (Table 3).

**Table 3:** Association of physical fitness parameters with hand grip strength.

Variables	30CST (no.)			6MWT (m)			2MWT (no.)			Sit-and-reach test (cm)			3MBWT (s)			F8WT (s)		
	*B*	SE	β	*B*	SE	β	*B*	SE	β	*B*	SE	Β	*B*	SE	β	*B*	SE	β
**Men**																		
HGS (kg)	0.249***	0.008	0.249	5.452***	0.679	0.290	0.961***	0.027	0.269	0.250***	0.012	0.166	− 0.067***	0.002	− 0.262	− 0.269***	0.007	− 0.267
Age (years)	− 0.303***	0.009	-0.258	− 5.851***	0.665	-0.318	− 0.898***	0.033	− 0.214	− 0.278***	0.014	− 0.158	0.089***	0.002	0.298	0.407***	0.009	0.346
BMI (kg/m^2^)	− 0.258***	0.016	-0.114	− 9.579***	1.369	-0.243	− 0.643***	0.059	− 0.080	− 0.342***	0.026	− 0.101	0.069***	0.004	0.120	0.315***	0.015	0.139
Constant	41.235***	0.865		1078.013***	71.036		158.782***	3.184		24.303***	1.376		− 0.053***	0.215		− 3.404**	0.824	
*R*^*2*^_*adj*_	0.177			0.248			0.159			0.075			0.216			0.261		
F(p)	1194.025			78.072			1008.199			451.281			1533.584			1967.716		
**Women**																		
HGS (kg)	0.303***	0.007	0.232	6.337***	0.544	0.251	1.349***	0.033	0.241	0.396***	0.010	0.230	− 0.098***	0.002	− 0.235	− 0.375***	0.008	− 0.243
Age (years)	− 0.269***	0.006	-0.247	− 5.494***	0.455	-0.259	− 1.270***	0.027	− 0.271	− 0.234***	0.009	− 0.163	0.130***	0.012	0.371	0.530***	0.007	0.411
BMI (kg/m^2^)	− 0.294***	0.010	-0.150	− 7.959***	0.645	-0.255	− 1.047***	0.046	− 0.124	− 0.291***	0.014	− 0.113	0.092***	0.003	0.146	0.358***	0.011	0.154
Constant	38.841***	0.581		1007.015***	40.718		192.301***	2.541		28.665***	0.791		− 2.921***	0.174		− 11.992***	0.621	
*R*^*2*^_*adj*_	0.179			0.214			0.195			0.119			0.280			0.327		
F(p)	2391.452			169.974			2240.888			1328.658			3826.315			4793.020		

*BMI* body mass index, *HGS* hand grip strength, *30CST* 30-s chair stand test, *6MWT* 6-min walk test, *2MWT* 2-min walk test, *3MBWT* 3-m backwards walk test, *F8WT* figure-of-8 walk test, *B* unstandardized regression coefficient, *SE* standard error, *β* standardized regression coefficient, *R*^*2*^_*adj*_ coefficient of determination.

***P < 0.001 through multiple linear regression analysis.

**Figure 2:**
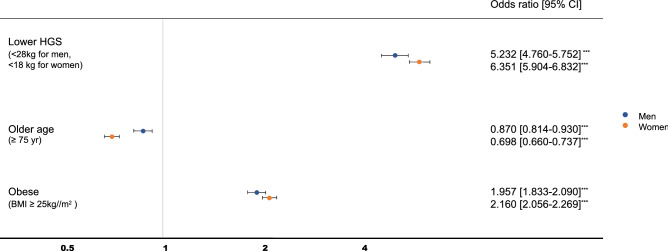
Odds ratio of the lowest level of overall physical fitness. Reference groups were those who were younger (< 75 years), non-obese (BMI < 25 kg/m^2^), and with higher HGS (≥ 28.0 kg for men and ≥ 18.0 kg for women). ^***^P < 0.001 through multiple logistic regression analysis. *HGS* hand grip strength, *BMI* body mass index, *CI* confidence interval.

Applying receiver operating characteristic (ROC) analysis, the optimal cut-off levels of HGS for having the lowest overall PF (< 30th percentile of any of the PF parameters based on the age- and sex-matched population) were 31.65 kg in men (area under the curve (AUC) 0.671, sensitivity 74.6%, and specificity 49.0%) and 19.95 kg in women (AUC) 0.677, sensitivity 79.7%, and specificity 53.0%). After adjusting for age and BMI, the groups with low HGS (< 28 kg for men and < 18 kg for women) had significantly higher odds of having the lowest level of overall PF (Fig. 2).

## Discussion

This study revealed the status of each PF parameter and the overall fitness level in healthy older adults using nationwide data in Korea. Additionally, muscular strength and endurance of the lower limbs, aerobic fitness, flexibility, balance, and coordination were estimated using HGS, and the lowest level of overall fitness was predicted with HGS.

The aging population is increasing worldwide, causing individual and social burdens, making physical fitness a public health concern. The prediction of lower limb strength, aerobic fitness, balance, and walking ability is essential even in healthy aging adults because they are closely related to their independent activities of daily living and quality of life^24–26^.

PF in Korean older adults has been measured using muscle strength and endurance (HGS and 30CST), aerobic fitness (6MWT or 2MWT), flexibility (sit-and-reach test), balance (3MBWT), and coordination (F8WT) in the NFA 100 projects, sufficient to reflect their mobility, ADL, and functional fitness^6^. The test–retest repeatability of each PF parameter in older adults was 0.619 for 3MBWT, 0.733 for 2MWT, 0.760 for 30CST, 0.88 for HGS, 0.99 for the sit-and-reach test, and 0.99 for F8WT in a previous report^6^, which was relatively low compared to all the results of the study of adults aged < 65 years, which was > 0.9^5^. While conducting PF tests in older people, the process can be time and effort exhausting, and physically unfit participants may experience fatigue and discomfort^8^. In addition, there may be some errors in the data or a lack of completion in the case of cognitive impairment.

The COVID-19 pandemic has significantly changed daily life. In the early pandemic stage, due to governmental policies, access to fitness testing centers was limited. Even after the reopening of the centers, some standard tests have been replaced temporarily by others that produce fewer droplets, and the obligation to wear a mask during the test increases the level of discomfort and inaccuracy in a participant’s result. We should now prepare for such situations when the measurement of PF is not readily available. Through this study, we aimed to assess whether a simple HGS test could estimate and predict other fitness parameters, such as muscular endurance and aerobic fitness, so it could eventually be used as a substitute for multiple tests, although it should be used only when the clinicians are not able to use gold standard methods.

In previous studies, significant correlations between HGS and PF tests were found in 30 college students performing the vertical and horizontal jump test, curl-up test, and VO_2max_^15^_,_ and in 54 college students performing the bent-knee sit-up test, push-up test, dumbbell swing, leg extension, and leg press test^27^. In older adults under institutional care, significant associations were found between HGS and lower limb strength and postural balance^28^. However, there have been few studies on the status of PF and the relationship between HGS and other PF parameters in healthy older adults from large nationwide data.

HGS is a simple, inexpensive, and useful method for measuring upper limb strength in limited time and space with many clinical values. It is associated with many other metabolic diseases^17–19,29–31^, frailty, and premature mortality^32–34^ since reduced muscle strength limits one’s activity and thus causes morbidities. Gait changes with age, mainly due to reduced lower limb strength, balance, and coordination, are known to be associated with risk of falling, functional decline, and dependence^35^. Based on the results of this study, HGS appears to be a viable substitute for many fitness tests in limited situations, and its measurement may therefore lead to early identification and interventions to prevent frailty, morbidity, and mortality in the high-risk group.

This study had some limitations especially due to its nature as a secondary analysis of an existing public dataset from the NFA 100. First, we could not evaluate other sociodemographic or nutritional factors that could influence one’s PF level. Second, the data did not include information about comorbidities or pharmacological treatments of the participants, which could be confounders of the results. Third, only data from older adults were included, and the results of this study cannot be applied to participants aged < 65 years. Further prospective studies including younger age groups should be performed after adjusting for other variables to improve the predictability of the results.

Nonetheless, since this study using large nationwide data revealed that HGS could estimate other PF parameters and overall fitness levels, HGS can be useful as a substitute test for PF in older adults. It could be helpful in predicting and preventing the functional decline of people who are physically unfit, very old, disabled, and have comorbidities, which highlights the value of HGS in public health.

In conclusion, we could estimate muscular strength and endurance, aerobic fitness, flexibility, balance, and coordination, and predict overall fitness level of Korean older adults using HGS. We also uncovered their status of PF levels from population-based data.

## Methods

### Data access and participants

**Figure 3:**
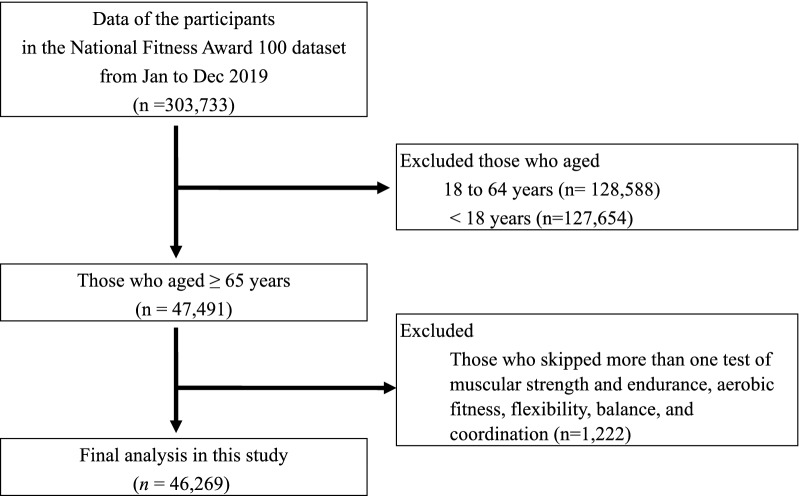
Flow chart of the sample analyzed in this study.

The NFA 100 dataset, a national project by the Korean Sports Promotion Foundation that assessed people’s PF in 76 centers nationwide, was used for the analysis in this study. Data were collected after measuring the determined PF parameters according to age groups using standardized protocols by certified instructors^4–6^. This NFA 100 dataset was acquired from the public platform which did not include participants’ personal information. Data of older adult participants aged ≥ 65 years obtained from January to December 2019 in the NFA 100 (n = 47,491) were included in this study. Data from participants who skipped more than one test of muscular strength and endurance, aerobic fitness, flexibility, balance, and coordination were excluded from the analysis (n = 1222). The values of outliers, considered as input errors, were treated as missing values. Finally, 46,269 participants (16,684 men and 29,585 women) were included in the statistical analysis (Fig. 3). This study was approved by the institutional review board of Pukyong National University (1041386-202112-HR-73-02), which waived the requirement for informed consent due to the retrospective design of the study.

### NFA 100 measurement protocol

#### Anthropometric measurement

Standing height was measured to the nearest 0.1 cm using a stadiometer (Seca Corporation, Columbia, MD, USA), and body weight was measured to the nearest 0.1 kg using bioelectrical impedance analysis (Biospace, Seoul, Korea). BMI was calculated as body weight (kg) divided by the square of height (m).

#### Muscular strength of the upper limb: hand grip strength (kg)^6^

HGS was used to measure the muscular strength of the upper limbs. It was measured using a Smedley-type hand dynamometer (Grip-D 5101; Takei, Niigata, Japan) in an upright position. The subjects were instructed to hold the dynamometer in the hand to be tested, with their arms straight and a 15° angle between the arm and body, and to squeeze the dynamometer with maximal isometric effort for 5 s. The handle of the dynamometer was located between the second interphalangeal joint of the four fingers and the palm at the base of the thumb. After measuring HGS twice, the highest value was recorded to the nearest 0.1 kg. The HGSs of the dominant and non-dominant hands were measured, but the results of the dominant hands were used for analysis.

#### Muscular strength and endurance of the lower limbs: 30-s chair stand test (number of times)^6^

The 30CST was used to assess muscular strength and endurance of the lower limbs. The subjects sat in the middle of the chair with their hands on the opposite shoulders crossed at the wrists and their feet flat on the floor. They were instructed to stand and sit completely at the start of the signal, which was repeated for 30 s. One measurement was allowed and the number of times they stood completely was recorded.

#### Aerobic fitness: 2-min walk test (number of times) or 6-min walk test (m)^6^

Aerobic fitness in was measured using either the 2- or 6-min walk test, depending on the measurement centers of the NFA 100. To measure the 6MWT, four cones were located in each corner of the 50-m rectangular track (20 m × 5 m) marked at intervals of 1 m from the starting line. For 6 min, the subjects were instructed to walk but not run on the track as quickly as possible at the start signal. They were allowed to rest in the middle, but the time continued. Only one measurement was performed to record the total distance walked. For the 2MWT, the midpoint of each participant’s thigh from the center of the patella to the iliac crest was marked with a piece of tape. A rubber band was then wrapped around two poles at the same height as the midpoint. Next, the participants were instructed to take steps in place and raise their knees until they touched the rubber band. If they did not reach the rubber band, the repetition was not counted. Only one measurement was performed to record the total number of steps with both feet.

#### Flexibility: sit-and-reach test (cm)^6^

The sit-and-reach test was used to assess flexibility. Participants sat on a mat with their knees fully extended and their soles touching the vertical board of a flexion meter (BS-FF; Biospace, Seoul, Korea). They were instructed to reach forward with their hands pushing out the ruler of the device as far as possible, without bending their knees. After two measurements, the highest value was recorded to the nearest 0.1 cm.

#### Balance: 3-m backward walk test (s)^6^

The 3MBWT was used to assess balance skills. A distance of 3 m between the front edge of a chair and the back of a cone was measured. Subjects were asked to sit in the middle of a chair with their back straight, feet flat on the floor, and both hands on their thighs. They were instructed to stand from the chair at the start of the signal, walk around the cone, return to the chair, and sit down as soon as possible. After measuring the time with a stopwatch twice, the fastest value was recorded to the nearest 0.1 s. Lower values indicate better balance skills.

#### Coordination: figure-of-8 walk test (s)^6^

The F8WT was used to assess coordination skills and evaluate walking ability and mobility. On the floor, a rectangle (3.5 m × 1.6 m) was marked with tape on all four corners, and cones were placed on the top two vertices. A chair was located at the midpoint of the bottom side of the rectangle with its back facing the two cones, measuring 2.4 m from each cone placed. Subjects were instructed to sit on the chair with their back to the cones, then at the start signal stand and turn around the cone on the right rear side, return and sit down on the chair. After measuring the time with a stopwatch twice, the fastest value was recorded to the nearest 0.1 s. Lower values indicated better coordination skills.

#### Overall PF level^6^

After adequate measurement of PF parameters of HGS, 30CST, 6MWT/2MWT, 3MBWT, F8WT and sit-and-reach test, the participants’ overall level of PF was graded from 1st to 4th level based on the grade of each parameter. The participants were accredited as 1st when all PF parameters were ≥ 70th percentile with age-and sex-matched norms, 2nd ≥ 50th percentile, 3rd ≥ 30th percentile. If any of the six PF parameters of the subjects were < 30th percentile, they were classified into the lowest overall fitness level group.

### Statistical analysis

Continuous and categorical variables were expressed as mean ± standard deviation and number (%). The demographic and anthropometric characteristics and PF parameters of the participants were analyzed using descriptive statistics. χ^2^-test was used to determine the difference in the proportion of overall fitness level according to sex and age groups. Relationships between HGS and other demographic and PF parameters were evaluated using Pearson’s correlation analysis. The strength of the correlation was considered poor, fair, moderately strong and very strong, if the Pearson’s correlation coefficients were 0.3, 0.3 to 0.5, 0.6 to 0.8, and > 0.8, respectively^36^. Multiple linear regression tests were performed to investigate the associations between HGS and other PF parameters after adjusting for age and BMI. ROC analysis was conducted to determine the optimal cut-off levels for HGS to detect the lowest overall PF, where the Youden index (sensitivity + specificity − 1) was maximized^37^. For multi-variated analysis, multiple logistic regression analysis was conducted to calculate the odds ratio of the lowest level of overall PF according to the HGS after adjusting for age and BMI. The groups who were younger (< 75 years), non-obese (BMI < 25 kg/m^2^), and with higher HGS were selected for the reference groups. For HGS, the cut-off values (< 28.0 kg for men and < 18.0 kg for women) by the Asian Working Group for Sarcopenia 2019 consensus^38^ were used, determined by the lowest quintile of the normative data from eight Asian cohorts^39^. The sensitivity and specificity to detect definite sarcopenia were 0.714 and 1.000 in men and 0.711 and 0.940 in women^40^. IBM SPSS (version 27; IBM Corp., Armonk, NY, USA) was used for analysis, and P*-*values < 0.05 were considered statistically significant.

## Data Availability

The datasets generated during and/or analyzed during the current study are openly available in the Bigdata Market C of Korea Culture Information Service Agency, https://www.bigdata-culture.kr/bigdata/user/data_market/detail.do?id=ace0aea7-5eee-48b9-b616-637365d665c1.
